# Spatial memory and frames of reference: How deeply do we rely on the body and the environment?

**DOI:** 10.1007/s10339-024-01211-2

**Published:** 2024-08-10

**Authors:** Tina Iachini

**Affiliations:** Laboratory of Cognitive Science and Immersive Virtual Reality, CS-IVR, Department of Psychology, University of Campania Luigi Vanvitelli, Caserta, Italy

## Abstract

How do we mentally represent the world out there? Psychology, philosophy and neuroscience have given two classical answers: as a living space where we act and perceive, dependent on our bodies; as an enduring physical space with its feature, independent of our bodily interactions. The first would be based on egocentric frames of reference anchored to the body, while the second on allocentric frames of reference centred on the environment itself or on objects. This raises some questions concerning how deep the reliance on the body and the environment is when using these reference frames, and whether they are affected differently by the duration of time and the scale (small or large) of space. To answer these questions, I have brought empirical evidence of the effect of motor interference, blindness, environmental characteristics and temporal factors on egocentric and allocentric spatial representational capacity. The results suggest that egocentric representations are deeply rooted in the body, with its sensory and motor properties, and are closely linked to acting *now* in small-scale or peripersonal space. Allocentric representations are more influenced by environmental than by bodily characteristics, by visual than by motor properties, and seem particularly related to large-scale or extrapersonal space. In line with neurophysiological evidence and a Kantian perspective, it appears that we are endowed with an internal spatial representation system ready to structure environmental information for our purposes. To what extent this system is innate and pervasive in cognition and what is its relationship to the neural 'positioning' substrate discovered by O'Keefe and colleagues requires further scientific investigation.

## Introduction

How do we represent the world out there? Psychology, philosophy and neuroscience have given two classical answers: as a living space where we act and perceive, dependent on our bodies; as an enduring and detached physical space, independent of our bodily interactions Eilan et al. 1993). This is consistent with a fundamental distinction in spatial cognition: egocentric and allocentric reference frames. Egocentric reference frames use the body or body parts as the anchorage of external positions and are dependent on the subjective vantage point (e.g. the object in front of you), while allocentric reference frames rely on external objects or on the environment itself (e.g. the object X on the right of object Y) and are independent of the subjective position (Kosslyn 1994; Milner and Goodale 1995, 2008; O’Keefe and Nadel 1978; Paillard 1991; Piaget and Inhelder 1967; Ruggiero et al. 2014). Neurofunctional studies have shown the activation of the posterior parietal/frontal network in egocentric processes and posteromedial and medio-temporal substructures in allocentric processes (e.g., Burgess 2006; Galati et al. 2010; Moffat 2009; Ruotolo et al. 2019). Furthermore, although a decline in spatial processing capacity has been reported during healthy age, it appears to be more marked in allocentric processing (Kirasic 1991; Lithfous et al. 2013; but see Lemay et al. 2004).

Thus, behavioral, neural and developmental data converge in supporting a distinction, at least partial, between egocentric and allocentric representations. However, the nature of these spatial frames has been long debated and has raised several questions such as: How deep is the reliance on the body and the environment when using these frames? Are they affected differently by time durations and scale (small or large) of space?

Since the hominids of the species Homo erectus, humans have explored the environment by interacting frontally with their surroundings. Therefore, because of their upright position relative to the ground, they have an egocentric interface with the environment. This led to a particular sensitivity to depth perspective and, as well as for other terrestrial animal species, a facilitation in localizing stimuli placed at various distances around the body (e.g. Delius and Hollard 1995). Allocentric representations would be orientation independent and an extra processing would be required to work out object-centred representations. Consistently, research has shown a facilitation (in terms of accuracy and response time) for egocentric representations over allocentric ones (Iachini and Ruggiero 2006; Iachini et al. 2014a; Millar 1994; Ruotolo et al. 2015; Ruggiero et al. 2016). However, spatial cognition is a multicomponent system that reflects several factors such as the grain of spatial relations, the role of motor sensory-resources and the range of space. In a series of studies, we explored whether motor interference, blindness, environmental characteristics and temporal factors can modulate the processing of egocentric and allocentric representations in combination with other factors. We adopted a simple “Ego-Allo task” where participants had first to learn triads of objects and then to retrieve their locations according to egocentric (e.g. where was the object closest to you?) and allocentric (e.g. where was the object closest to a target?) reference frames (Iachini and Ruggiero 2006). The results of decades of studies showed that several factors combine and nuance spatial processing, thus shedding light on the very nature of basic reference frames.

## Grain of spatial relations and temporal factors

We can specify the grain of spatial relationships at different levels of precision ranging from metrically precise to more abstract. According to Kosslyn (1994), coordinate relations specify precise spatial locations in terms of exact metrics, whereas categorical relations form general, abstract and invariant spatial information such as left/right, above/ below. This distinction is supported by convergent data (Ruotolo et al. 2019; Ruggiero et al. 2014; Ruotolo et al. 2011; Ruotolo et al. 2015; for reviews Colombo et al. 2017; Postma et al. 2008).

In two behavioural studies, Ruotolo and colleagues explored the effect of temporal delay (1.5 vs. 5 s), stimuli characteristics (3D objects vs. 2D images) and motor (Ruotolo et al. 2015) vs verbal (Ruotolo et al. 2016) response modalities on the combination of frames of reference and spatial relations. After learning the position of objects, participants had to judge the distance (coordinate) and the relation (categorical) of a target object with respect to themselves (ego) or another object (allo). A specific effect of duration appeared: coordinated egocentric judgements were favoured with an immediate motor response on 3D-manipulable stimuli, whereas allocentric and categorical judgements were favoured with a delayed verbal response on 2D-non-manipulable stimuli. These results are consistent with Kosslyn (1994) and Milner and Goodale’s “perception–action” model (Milner and Goodale 1995, 2008; see also Iachini et al. 2009) suggesting that categorical and allocentric processing is mainly involved in long-term object recognition, whereas coordinate and egocentric processing in immediate action. Thus, spatial representations are strongly anchored to the body in tasks with visuomotor rather than visuo-perceptual features.

## Grain of spatial relations and motor resources in peripersonal and extrapersonal spaces

A long tradition in western thought that has proposed that the experience of spatiality would originate from action, that is from representing the movements that are necessary to reach a position (e.g., Merleau-Ponty 1945; Poincaré 1902). Modern neurocognitive approaches assume that external space involves perceptual thresholds defined by their relevance for action. The area of space around our body, within the reach of our limbs and where multisensory signals are integrated egocentrically for acting in the *here and now*, is called peripersonal space (e.g., Berti and Frassinetti 2000; Rizzolatti and Matelli 2003; Ruggiero et al. 2014). A dedicated frontal-parietal network would subtend the processing of this space (e.g. Farnè et al. 2005). This area is defined as an action space for body–objects interactions (Rizzolatti et al. 1997; Stein and Meredith 1993) and as a defensive space to preserve body integrity (Graziano 2017; Iachini et al. 2014a, b; Ruggiero et al. 2021a, b). Instead, extrapersonal space refers to the area beyond reaching where we cannot act in the immediacy. This far space seems more linked to visual–spatial mechanisms and is mainly processed through the ventral visual stream (Bartolo et al. 2014).

Iachini and colleagues (2009) compared Right-Brain-Damaged (RBD) and Left-Brain-Damaged (LBD) patients (not affected by neglect or language disturbances) with normal controls to verify whether lateralized parietal brain lesions may selectively impair egocentric and allocentric processing of spatial information in near/far spaces. LBD patients presented difficulties in both egocentric and allocentric processing, whereas RBD patients were impaired in egocentric judgements, and in near but not far space. This suggests that the right hemisphere is more specialized in processing egocentric frames of reference in peripersonal space. A recent study Confirmed that the encoding of peripersonal space involves motor processes per se and not because of the presence of manipulable stimuli (Iachini et al. 2014b). Indeed, participants were faster and more accurate in localizing all kinds of stimuli in peripersonal (not extrapersonal) space when motor resources were fully available, not interfered. The selective effect of action possibility could reflect an evolutionary pressure: reacting in time to events near the body by anticipating what could happen in the next. This might also have made humans highly sensitive to dynamic temporal information for moving stimuli within peripersonal space (Iachini et al. 2017).

A recent study explored the role of motor resources on the combinations of three visuo-spatial factors: egocentric and allocentric frames of reference, categorical and coordinate spatial relations, peripersonal and extrapersonal space (Ruggiero et al. 2020). While participants had their arms free or blocked (motor interference), they had to verbally provide spatial judgments combining frames of reference and spatial relations about memorized objects placed in peripersonal or extrapersonal spaces. Motor interference had no effect on extrapersonal space but impaired exclusively egocentric-coordinate judgments in peripersonal space. This combination seems particularly important in planning and performing reaching movements (e.g. Tipper et al. 1992), and in supporting heading orientation (Ciaramelli et al. 2010; Ruggiero et al. 2014). We may conclude that the visuo-spatial memory components necessary to localize spatial stimuli include several abilities along an ideal continuum, with frames of reference, spatial relations and peri/extrapersonal space mechanisms cooperating to organize spatial information for perceptual-based and action-based purposes.

## Blindness and frames of reference

While several findings suggest that vision is the dominant perceptual modality in spatial cognition, others suggest that vision is neither sufficient nor even necessary to form adequate spatial mental representation (e.g. Millar 1994; Pasqualotto and Proulx 2012; Rieser et al. 1986; for reviews Thinus-Blanc and Gaunet 1997; Cattaneo et al. 2008). Nevertheless, early visual input should be fundamental in setting up the cerebral architecture underlying spatial functions (Cohen et al. 1997; Maurer et al. 2005). Much evidence shows that congenitally blind people perform egocentric tasks similarly to sighted individuals, but perform worse in allocentric tasks (Cattaneo et al. 2008; Merabet and Pascual-Leon 2010; Millar 1994; Thinus-Blanc and Gaunet 1997). Moreover, spatial tasks based on locomotor exploration of a large-scale space seem more difficult for congenitally blind participants than late blind and sighted participants (Rieser et al. 1986; but see Loomis et al. 1993).

Given these premises, Iachini and colleagues investigated whether the influence of the visual status on egocentric and allocentric spatial representations is modulated by the scale of space (Iachini et al. 2014a) and aging (Ruggiero et al. 2021a, b). Results showed that congenitally blind people struggled more with allocentric representations, especially in large spaces where they need to explore by touch and movement. Furthermore, aging worsened allocentric abilities, especially in those who were born blind. These findings demonstrate a decremental effect of concurrent aging and congenital blindness on the ability to represent spatial relationships between external, environment-centred anchor points.

In daily life we constantly switch from one reference frame to another, such as when we have to to grasp a pen (egocentric) between a book and a cup (allocentric). Ruggiero et al. (2018a, b) compared congenitally blind, sighted and blindfolded sighted participants on the capacity to provide spatial judgments in switching (from ego-to-allo, from allo-to-ego) and non-switching (only-ego, only-allo) conditions (“Ego-Allo Switching Task”). Congenitally blind participants showed a general allocentric difficulty and, importantly, a specific difficulty in switching from allocentric to egocentric representations. Instead, when the first anchor point was egocentric, no difference between groups appeared.

In conclusion, early visual experience contributes importantly to develop accurate allocentric representations, especially of large-scale spaces. This is probably due to the capacity of vision to encode external spatial positions simultaneously, while alternative perceptual modalities (e.g., proprioceptive, tactile) require time-consuming and sequential exploratory movements centered on the body. Indeed, when sighted people are forced to encode spatial information in a sequential way to build up a spatial map, their performance is similar to that of congenitally blind people (Ruotolo et al. 2012). Thus, the facilitation of the egocentric processing, especially in small-scale space, would be rooted on the body that offers a stable anchor point even in the absence of vision.

## Environmental characteristics and frames of reference

According to McNamara and colleagues, it is important to consider the whole structure of an environment, such as the walls of a room (global reference frame) or a table (local reference frame). Spatial memory is facilitated when such environmental axes are aligned rather than misaligned with the egocentric learning view (Avraamides and Kelly 2008; McNamara 2003; Kelly and McNamara 2008). This raises the question of whether aligned/misaligned environmental axes influence the switching process. Orti et al. (2023), by using Immersive Virtual Reality, manipulated the orientation of the walls that could be either parallel (aligned) or rotated (misaligned) with respect to a table containing objects (the egocentric perspective). After learning, participants provided spatial judgments in switching and non-switching conditions. The results were clear: misaligned environments made it harder not only to remember object locations especially when using an allocentric frame, but also to switch from an allocentric to an egocentric frame. Thus, a misalignment between body and environment disrupts not only the allocentric component per se but also the egocentric component immediately following it. This reinforces the idea that the starting anchor point has a fundamental role (for similar results in pathological aging: Ruggiero et al. 2018a, b).

Further research used fNIRS to understand the brain activity involved in spatial switching (Orti et al. 2024). The results showed increased cortical activity in the temporo-parietal junction during the switching condition compared to the non-switching condition. This junction could be involved early in switching between body- and object-centred frames of reference, probably due to the preparatory role of this brain region in the pre-allocation of the cognitive resources required for switching processes. These results are consistent with the visuospatial memory model (i.e., the "two-system model") proposed by Burgess and colleagues (Burgess 2006; Byrne et al. 2007), according to which egocentric and allocentric spatial representations cooperate.

## Conclusions

In sum, egocentric representations are deeply rooted in the body, with its sensory and motor properties, and are closely linked to acting *now* in small-scale or peripersonal space. Allocentric representations are more influenced by environmental than bodily characteristics, visual than motor properties, are more enduring and seem particularly related to planning in large-scale or extrapersonal space. Our results show that the two egocentric and allocentric systems cooperate, are present at different levels of involvement in a wide range of activities from recognition to action. From a Kantian perspective and in line with neurophysiological evidence, it appears that we are endowed with an internal spatial representation system ready to structure environmental information for our purposes. Behavioral and neural data suggest partial functional and neuroanatomical overlaps that evidently reflect some common mechanism underlying the two reference frames. However, functional specializations also emerge that are flexibly modulated by needs and purposes in interaction with the environment and are also reflected in specific neural activations. It is possible that through learning we do not draw up a "code" or "neural metrics" to represent physical space (O'Keefe 1993). Rather, it is possible that we use this basic "neural space" to construct specialized spatial representations that best support our needs and purposes in daily life. To what extent this system is innate and pervasive in cognition and what is its relationship to the neural 'positioning' substrate discovered by O'Keefe and colleagues requires further scientific investigation.
